# Early Perception of Intonation in Down Syndrome: Implications for Language Intervention

**DOI:** 10.3390/ejihpe15100194

**Published:** 2025-09-26

**Authors:** Cátia Severino, Marina Vigário, Sónia Frota

**Affiliations:** Center of Linguistics, School of Arts and Humanities, University of Lisbon, 1600-214 Lisbon, Portugal; catiaseverino@edu.ulisboa.pt (C.S.); mvigario@edu.ulisboa.pt (M.V.)

**Keywords:** Down Syndrome, infants, intonation, speech perception, early language abilities, visual habituation, prosody

## Abstract

Language difficulties have been highlighted as a cornerstone of the developmental profile in Down Syndrome (DS), but very few studies have examined early language abilities in children with DS to determine the initial strengths and weaknesses that might inform early language interventions to support language development in this population. This study focused on the early perception of intonation and examined whether it differed between infants with DS and typically developing (TD) peers. Using a visual habituation paradigm from a previous study on TD infants’ ability to perceive the intonation of statements and questions, infants with DS were able to successfully discriminate statement and question intonation, similarly to TD infants. However, unlike for TD infants, an age group effect was found, with older infants with DS being unable to discriminate the intonation contrast. Our findings highlight the importance of prosody in early development also in infants with DS. Moreover, the unexpected decrease in early sensitivity to intonation in older infants with DS pinpoints a crucial developmental window—the first semester of life—for early interventions using intonation to support language learning in these infants.

## 1. Introduction

Down Syndrome (DS), a genetic perturbation that results from a partial or complete duplication of chromosome 21, is the most common genetic cause of intellectual disability, with an incidence between 1 in 319 and 1 in 1000 live births (Asim et al., 2015; Ilyas et al., 2020). Children with Down Syndrome display a complex neurocognitive profile that includes both language comprehension and language production deficits, affecting language development and language processing (Chapman, 1997; Finestack et al., 2013; Kent & Vorperian, 2013; McDuffie & Abbeduto, 2009). Indeed, language has been reported to be one of the most impaired domains in individuals with Down Syndrome, with long lasting consequences for their inclusion in society (Abbeduto et al., 2007; Buckley, 1993; Martin et al., 2009). Despite the widely recognized importance of language, few studies have investigated early language abilities in infants with DS. Therefore, it is largely unknown what their initial language strengths and weaknesses are. Such information is of the utmost importance to inform early language intervention programs to support language development in this clinical population.

The present study contributes to filling this research gap by focusing on the early perception of intonation in infants with DS. Intonation appears to be a universal property of language, in the sense that all languages employ intonation (Best, 2019; Gussenhoven, 2004). Intonation is the domain of prosody that refers to the use of the speech features of pitch, duration and intensity to convey phrase and sentence-level meanings that are independent of the word meanings (Gussenhoven, 2015; Ladd, 2008). A well-known example of sentence-level meanings that are conveyed by intonation in many languages is the contrast between a statement and a question (Gussenhoven, 2016): the former is usually expressed by a falling pitch, whereas the latter is usually expressed by a rising pitch. The goal of the current study is to investigate the development of early perception abilities for intonation in Down Syndrome and examine whether the development of these abilities differs between infants with DS and their typically developing (TD) peers. To probe early perception abilities, the intonation contrast between statements and questions was used. If infants with DS display poor discrimination abilities for intonation, the domain of intonation will be a further domain of difficulties that needs to be addressed in early language intervention programs. If, by contrast, early discrimination abilities for intonation constitute an initial strength, intonation might be used in early intervention as a tool to boost language learning, given that many language categories and functions are cued by intonation.

## 2. Background

Early sensitivity to prosody is well documented in typically developing infants (e.g., Jusczyk, 1997; Nazzi et al., 1998a, 1998b; Sansavini et al., 1997; for recent reviews, Frota & Butler, 2018; Liu et al., 2022). In the intrauterine environment, the speech sound information available to the fetus is greatly reduced, but intonation is almost perfectly reproduced in utero (Querleu et al., 1988). Soon after birth, infants are sensitive to speech rhythm, prominence patterns and differences in pitch contours, showing that newborns can recognize the speech features of pitch, duration and intensity that are used in intonation. When these prosodic properties are impoverished through artificial manipulations of speech, newborns show decreased brain responses compared to those found when listening to natural speech prosody (Sambeth et al., 2008). Additionally, newborns seem to be more sensitive to the prosodic patterns present in the language(s) they were exposed to before birth. For example, newborns born to monolingual French-speaking families are more sensitive to duration contrasts, whereas newborns born to bilingual families (speaking French and another language) are more sensitive to pitch contrasts (Abboub et al., 2016). These findings suggest that neonates already distinguish between pitch, duration and intensity patterns they are more familiar with and those they are unfamiliar with, since they have not been exposed to or have been less exposed to such patterns prenatally.

Research has shown that this early sensitivity to prosody enables infants to exploit the pitch, duration and intensity cues available in speech to learn about many different properties of their native language(s) (e.g., Gervain et al., 2020). For example, to learn words infants need to identify and extract words from the continuous speech they hear. This is a challenging task that they approach by first segmenting words that display salient prosodic cues, such as high pitch, large pitch movement and longer duration. American English-learning infants as young as 6 months were able to segment target words from continuous speech only if they exhibited these prominent pitch features (Johnson et al., 2014). Similarly, European Portuguese-learning infants were able to segment target words that showed these prosodically salient features from 4 months of age, but at 10 months were still struggling to segment non-prosodically prominent target words (Butler & Frota, 2018). Intonation has also been shown to guide German-learning infants’ ability to segment words, given that they perceive syllables with high pitch as signalling the beginning of a word (Zahner et al., 2016). Furthermore, 6-month-old infants have been shown to map a segmented target word into a visual referent (which is a precursor to word learning) only when the target matched a cohesive intonation unit (Shukla et al., 2011). Thus, prosody, and intonation in particular, facilitates early word segmentation and, consequently, promotes word learning.

The finding that typically developing children use prosody, intonation included, to learn about the grammar and lexicon of their native language has highlighted the need to investigate prosodic abilities in clinical populations (Goswami, 2022; Paul et al., 2020; Peppé, 2018) and to develop prosodic interventions to support language learning (Hargrove, 2013; Mandke et al., 2023).

Language learning difficulties in children with DS are manifested by the late occurrence of first words/signs (close or after the second birthday) and the delayed acquisition of native language sound patterns (Stoel-Gammon, 2001). Prelinguistic communicative gestures are also rarely paired with vocalizations (Greenwald & Leonard, 1979). In general, expressive language abilities are delayed, including vocabulary and syntax (Kover et al., 2012), and growth slopes in comprehension tend to become shallower with age (Kent & Vorperian, 2013). Importantly, early vocalizations and vocabulary level have been found to be early predictors of language development trajectories in DS.

However, very few studies have examined prosodic development in DS. Stojanovik (2011) showed that both the comprehension and production of prosody are impaired in children with DS between 8 and 12 years of age. Filipe et al. (2023) reported a similar finding for toddlers with DS. Mason-Apps et al. (2018) demonstrated that the development of word segmentation abilities in 18–20-month-old toddlers with DS is seriously delayed, and Frota et al. (2020) found that infants and toddlers with DS display difficulties in using intonation as a facilitator for word segmentation, unlike TD infants. However, the children with DS that were better at using intonation to segment words showed better expressive vocabulary scores. To our knowledge, there are no studies on the prenatal or neonatal perception of intonation conducted on Down Syndrome. Therefore, early perception abilities for prosody in DS have not yet been investigated in detail. It is thus unknown whether infants with DS are generally less sensitive to speech rhythm, prominence and/or intonation, and thus less able to exploit pitch, duration and intensity cues to learn the language, or whether certain aspects of prosody constitute initial strengths that might help them with language learning. For typically developing children, early language perception abilities have been shown to play a fundamental role in language acquisition, with cascading effects on later word learning and syntactic processing skills (Werker, 2018). Understanding how these early abilities develop in DS is thus critical for the early planning of intervention strategies to promote language learning in this clinical group.

The current study examines the perception of intonation in European Portuguese (EP)-learning infants with DS between 5 and 13 months of age. We followed up on Frota et al. (2014), who studied early perception of intonation in EP-learning TD infants between 5 and 9 months of age. Frota et al. (2014) assessed the discrimination of the statement/yes–no question contrast, which is conveyed solely by prosodic means in EP. For example, and unlike in English, the statement ‘Chegou.’ (‘He has arrived.’) and the yes–no question ‘Chegou?’ (‘Has he arrived?’) are distinguished only by prosodic cues. Specifically, the two sentence types are differentiated by intonation, with statements showing a falling pitch movement followed by a low pitch, and questions displaying a falling pitch movement followed by a rising pitch. Besides these crucial pitch cues, questions also show a longer duration than statements (Frota, 2002). The statement/question intonation contrast has been shown to be perceived by adult native speakers (Falé & Hub Faria, 2005).

Using a modified version of the visual habituation paradigm and stimuli consisting of one-word utterances with varying consonants and vowels, Frota et al. (2014) demonstrated that TD infants were able to successfully discriminate statements and yes–no questions as early as 5 months of age. Two groups of infants were compared, a younger group (5–6 months) and an older group (8–9 months), and no differences were found across groups. Infants are thus sensitive to the intonation cues that signal the sentence type distinction very early on in development, and were shown to maintain this sensitivity during the first year of life. The precocious discrimination abilities for intonation across segmental variability in consonants and vowels indicate that infants have the ability to extract and generalize the contrastive intonation patterns to different and new one-word utterances. Given that the statement/question distinction in EP is solely cued by intonation, the ability to perceive the differing intonational patterns is a prerequisite for the acquisition of sentence type distinction, and may thus facilitate the learning of the two sentence type categories. Notably, the categories of statement and question constitute basic and frequent sentence types in speech, which are key for communication and social interactions (Koegel et al., 2010; Newport et al., 1977). The acquisition of these categories is thus important to speech processing, language learning and social communication.

To determine whether EP-learning infants were responding to phonetically salient contrasts irrespective of their native language, or whether their sensitivity to the intonational contrast was modulated by the language they were exposed to, the same EP statement/yes–no question distinction was presented to Basque-learning, English-learning and German-learning TD infants (Sundara et al., 2015; Sundara & Frota, 2025; Czeke et al., 2019). In all these languages, prosodic cues are used to mark the sentence type distinction, but with language-particular differences. Moreover, while EP and Basque only employ intonation, English and German use both word order (i.e., an auxiliary verb before the subject in questions) and intonation. These studies showed that English- and German-learning infants had difficulties in discriminating the intonation contrast, whereas Basque-learning infants successfully distinguished the statement and question patterns, like their EP-learning peers. The findings thus suggest that the ability to discriminate the intonation contrast is influenced by the specific properties of the native languages, possibly related to the nature, distribution and/or the role played by intonation in signalling the sentence type meaning distinction. In other words, infants’ (lack of) sensitivity to the statement/question contrast is modulated by their early language experience as young learners.

If EP-learning infants with DS demonstrate similar perception abilities as EP-learning TD infants, discrimination of the intonation distinction will be successful, and infants with DS will show a similar sensitivity to the intonation contrast and a similar ability to extract and generalize the intonation patterns across phonetic variability. Such a result would indicate that intonation might be viewed as a facilitator for language learning in this clinical population. If, by contrast, EP-learning infants with DS fail to discriminate the salient statement/yes–no question distinction, this would indicate that intonation is a domain of difficulties that is manifested quite early in development, with consequences for the learning of the native language. The first outcome would support the use of intonation in early intervention as a tool to boost language learning, by exploiting the intonational cues to learn the lexical, syntactic and pragmatic language properties with which those cues are correlated. The second outcome would signal intonation as a weakness to be targeted in early intervention. Any of the outcomes will contribute to a better understanding of how early prosodic development, and language development in general, unfolds in children with DS, with implications for language intervention to support language learning in this population.

## 3. Materials and Methods

The intonation discrimination experiment from Frota et al. (2014) was used. The stimuli, paradigm and testing procedure were the same as in the original study. The study was conducted in accordance with the recommendations of the European Union Agency for Fundamental Rights and the Declaration of Helsinki, and approval was obtained by the Ethical Committee for Research of the School of Arts and Humanities of the University of Lisbon (H21 project, 1_CEI2018; P2LINK project, 19_CEI2021). Infants’ parents gave written informed consent before participation in the study.

### 3.1. Participants

Twenty-three infants with DS participated in this study (eleven girls, mean age 8 months and 2 days, age range from 5 months and 3 days to 13 months and 11 days). All participants were raised in monolingual European Portuguese-speaking homes and were recruited from collaborating clinical institutions. They were born full-term and had normal hearing to mild hearing loss (according to clinical screening). Another infant with DS was tested but excluded from analysis due to age (19 months). Although there is no data on the prevalence of DS in Portugal, the National Registry of Chromosome abnormalities (INSA) estimates that about 320 infants with DS have been born in Portugal (live births) between 2008 and 2017. Therefore, our sample is estimated to include around 20% of the infant population with DS in Portugal.

### 3.2. Stimuli

The stimuli from Frota et al. (2014) were used. It consisted of a set of 16 bisyllabic pseudo-words. All pseudo-words were CVCV sonorant sequences with stress on the initial syllable (malo, lemo, loma, mela, rono, rano, nurra, nirra, lamo, milo, mola, luma, norro, reno, nerra, rina). The pseudo-words were produced either as one-word statements or yes–no questions by a female native speaker of EP in child-directed speech. A detailed acoustic analysis of the stimuli showed the expected differences in pitch range (with larger pitch range in questions: mean range of 25 Hz in statements versus 192 Hz in questions) and final pitch height (with lower pitch in statements, mean 163 Hz and higher pitch in questions, mean 380 Hz). In addition, questions also showed longer duration (mean 765 ms versus 529 ms for statements), given the need to accommodate the complex falling–rising pitch movement and the larger pitch range.

Four sound files with eight one-word utterances were built, two for statements and two for questions. Each sound file had a total duration of 16 s, with an average interstimulus interval of 1471 ms for the statement files and 1235 ms for the question files. Different sound files, and thus different pseudo-words, were used in the habituation phase and test phase of the experiment. The stimuli are available at http://labfon.letras.ulisboa.pt/babylab/Infants_Perception/Infants_perception_intonation_supporting_materials.htm (accessed on 7 April 2025).

### 3.3. Procedure

**Figure 1:**
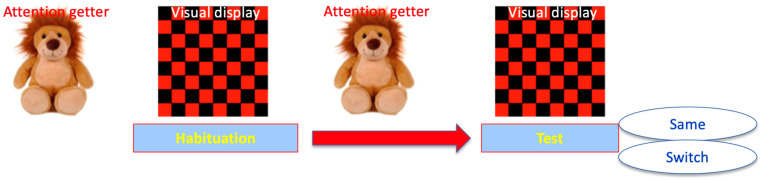
Experimental design: Implementation of the visual habituation paradigm.

As in Frota et al. (2014), a modified version of the visual habituation paradigm was employed. This is illustrated in Figure 1. Infants were comfortably seated on their caregivers’ lap in front of a computer monitor. A trial started with a colourful, attractive image as an attention getter. After fixating on the image for 2 s, infants were presented with a red and black checkerboard display, together with the habituation stimuli delivered through speakers discreetly positioned behind the monitor. The trial continued until the infant looked away from the screen for more than 2 consecutive seconds or the end of the sound file was reached. Then, the image reverted to the attractive attention getter and the next trial initiated. Stimuli presentation was controlled using dedicated visual habituation software, with an experimenter monitoring the infants’ orientation to the screen via a camera positioned above the monitor. The experimenter wore headphones playing music to mask the experiment sounds and remained blind to the experimental conditions while online coding infants’ looking time by pressing a computer button. Habituation trials continued until a predetermined criterion was met, defined as the average looking time at the screen for the last four habituation trials being less than 65% of the average looking time for the first four habituation trials. Following habituation, infants were presented with two test trials: one ‘same’ (as the habituation) and one ‘switch’ (different from the habituation). Half of the infants habituated to statement intonation and the other half to question intonation, and the order of presentation of test trials was counterbalanced across infants.

Eight infants had their looking times recorded and monitored using the LOOK software (Meints & Woodford, 2008), and fifteen infants had their looking times recorded and monitored using the Habit2 software v.2.2.10 (Oakes et al., 2019). The shift in software was necessary given the limitations in the compatibility of the LOOK software with more recent operating systems. The two software present stimuli and record and monitor looking times in similar ways.

If infants with DS were sensitive to the intonation contrast between statements and yes–no questions, they should exhibit longer looking times towards the ‘switch’ trials, like their TD peers (Frota et al., 2014), indicating successful discrimination of the two sentence types.

## 4. Results

All analyses were performed using SPSS v 29.0.2.0(20) (IBM Corp., 2024).

### 4.1. Infants with Down Syndrome (DS)

We first replicated the analysis in (Frota et al., 2014) for the infants with DS.

**Figure 2:**
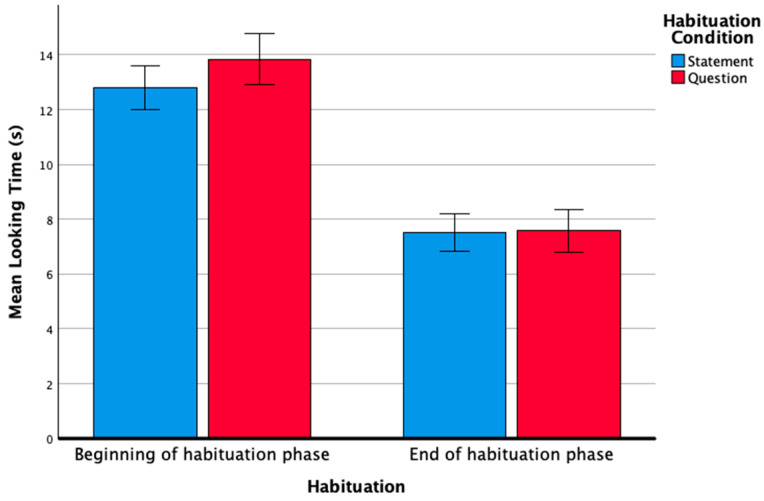
Average looking times (in seconds) for beginning and end of habituation. Error bars indicate the standard error of the mean.

Infants with DS exhibited a longer average looking time in the beginning of the habituation phase, i.e., the first four trials (*M* = 13.25, *SD* = 2.91), than in the end of the habituation phase, i.e., the last four trials (*M* = 7.54, *SD* = 2.40) (Figure 2). A repeated measures analysis of variance (ANOVA) was conducted on the average looking times in the first four and last four habituation trials, with a within-subject factor of habituation (beginning versus end of habituation phase) and a between-subject of habituation condition (statement versus question). The results revealed a significant effect of habituation (*F*(1, 21) = 72.711, *p* < 0.001, *η*^2^*_p_* = 0.77), but no significant effect of the type of habituation condition (*F*(1, 21) = 0.368, *p* = 0.551, *η*^2^*_p_* = 0.017), and no significant interaction (*F*(1, 21) = 0.512, *p* = 0.482, *η*^2^*_p_* = 0.024). Thus, infants with DS performed similarly in habituating to the stimuli, independently of the habituation condition.

**Figure 3:**
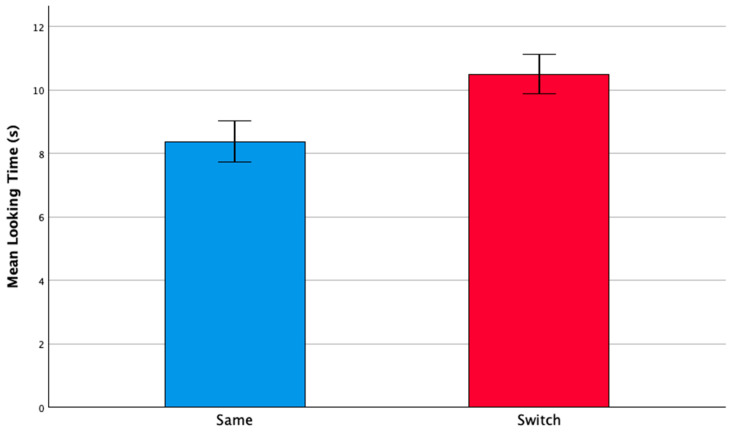
Average looking times (in seconds) for same and switch test trials. Error bars indicate the standard error of the mean.

In the test phase of the experiment, infants with DS exhibited longer average looking times for switch trials when compared to same trials (same *M* = 8.38, *SD* = 3.08; switch *M* = 10.51, *SD* = 2.96), as shown in Figure 3. A repeated measures ANOVA, with a within-subject factor of trial type (same versus switch) and a between-subject factor of habituation condition (statement versus question), revealed a significant effect of trial type only (*F*(1, 21) = 5.922, *p* = 0.024, *η*^2^*_p_* = 0.220). Neither the main effect of the habituation condition (*F*(1, 21) = 1.142, *p* = 0.297, *η*^2^*_p_* = 0.052) nor the interaction (*F*(1, 21) = 0.417, *p* = 0.526, *η*^2^*_p_* = 0.019) were significant. Further analysis using paired t-tests indicated significant differences between the same and switch trials (*t*(22) = 2.574, *p* = 0.017, *d* = 0.54, 95% *CI* −3.83, −0.413), with participants displaying longer looking times for switch trials.

These results follow the same pattern, in both the habituation and test phases, as the findings reported in Frota et al. (2014). The results thus indicate that infants with DS are able to successfully discriminate utterances that only differed in statement and question intonation, similarly to typically developing infants. In other words, infants with DS are sensitive to key prosodic differences in their native language. However, there seems to be more variability in the data of the infants with DS than in the TD infants’ data: 61% of the infants with DS showed longer looking times to the switch trials, compared to 88% of the TD infants. This difference is reflected in the strength of the effect sizes for the trial type factor, with a smaller effect size for infants with DS (*η*^2^*_p_* = 0.220) than for TD infants (*η*^2^*_p_* = 0.601).

### 4.2. Infants with Down Syndrome (DS) Versus Typically Developing (TD) Infants

To directly compare the results between infants with DS and TD infants, we used the data from the forty TD infants who participated in the study of Frota et al. (2014), available at http://labfon.letras.ulisboa.pt/babylab/Infants_Perception/Infants_perception_intonation_supporting_materials.htm (accessed on 7 April 2025). In addition, to explore potential age effects along the lines of Frota et al. (2014), we also divided infants with DS into a younger group (below 7 months of age; N = 13, mean age = 5 month 25 days, age range 5 months 3 days to 6 months 16 days) and an older group (above 7 months of age; N = 10, mean age 11 months, age range 7 months 3 days to 13 months 11 days). There was no age difference between the younger DS group and the younger TD group (younger TD, mean age 5 months 29 days; *t*(31) = 0.471, *p* = 0.641). However, the two older age groups were different, with the DS group being older than the TD group (older TD group, mean age 8 months 12 days; *t*(28) = 3.574, *p* = 0.005).

#### 4.2.1. Analysis of the Habituation Phase Data

First, we examined potential differences across the number of trials infants needed to habituate to the intonation pattern (statement or question) they were exposed to. A Mann–Whitney U test was conducted to compare DS and TD, revealing no significant differences between the two groups (*U* = 405.50, *z* = −0.78, *p* = 0.433, *r* = 0.10). The median number of trials for habituation was 11.00 for infants with DS and 10.00 for TD. Then, we examined possible differences across age groups (younger, older) and group types (DS, TD). A Kruskal–Wallis H test showed no significant differences (*H*(3) = 1.71, *p* = 0.635, *ε*^2^ = 0.00). Pairwise comparisons, corrected for multiple tests, confirmed the absence of differences between groups (all *ps* > 1).

We also examined possible differences in looking times for habituation trials. In Frota et al. (2014), while no differences were reported between the younger and older groups of TD infants for the number of trials needed to habituate, the looking times at the beginning of habituation (first four trials) were found to differ between the younger infants (with longer looking times) and older infants (with shorter looking times). The authors suggested that these results were indicators of differing levels of attention, with older infants displaying overall less attention in the task. We compared the looking times at the beginning of habituation between younger and older infants with DS. The Mann–Whitney U test indicated no significant difference in average looking times at the beginning of habituation between the younger (*Mdn* = 14.23) and older (*Mdn* = 14.25) age groups (*U* = 63.00, *z* = −0.124, *p* = 0.901, *r* = 0.03). Importantly, the looking times of both age groups of infants with DS were closer to those displayed by younger TD infants (*Mdn* = 13.81) than those displayed by older TD infants (*Mdn* = 12.09). The fact that older infants with DS behaved like younger TD infants suggests that they display more attention to process intonation, unlike older TD infants.

#### 4.2.2. Analysis of the Test Phase Data

**Figure 4:**
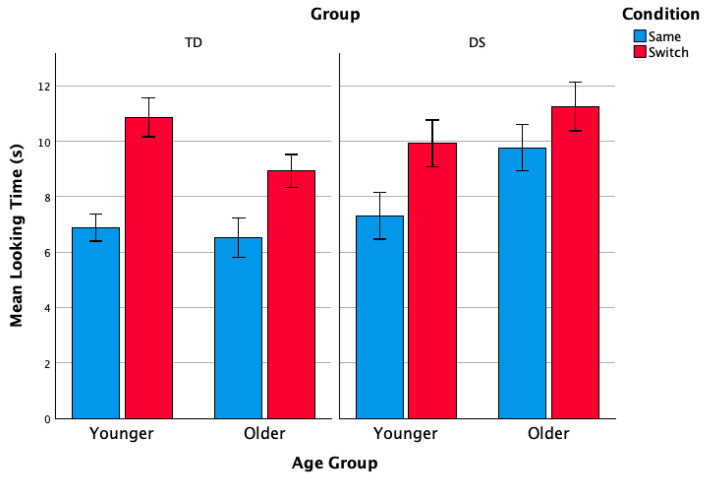
Average looking times (in seconds) for same and switch test trials by age group for both TD infants and infants with DS. Error bars indicate the standard error of the mean.

The average looking times for the same and switch test trials for both TD and DS by age group are presented in Figure 4. To compare the looking times of TD infants and infants with DS for the test trials, we used a generalized linear mixed model analysis (GLLM). Given the findings from the habituation phase, and to explore potential age group effects during the test phase, we included age group as a factor in the model. The data structure was defined based on participants. We included trial type (same, switch), group (TD, DS) and age group (younger, older) as fixed effects, along with the interactions between trial type and group, trial type and age group, and trial type, group and age group. Participant was included as a random effect (by-subject intercept). We used the Satterthwaite approximation method to account for differences across groups, and a robust estimation of the covariances to account for small sample sizes. Results are given in Table 1.

The analysis showed a significant effect of trial type, as expected, as both groups exhibited longer looking times towards the switch trials (switch *M* = 10.254, *SE* = 0.364; same *M* = 7.632, *SE* = 0.355). There were no effects of group or age group, although group approached significance, with infants with DS (*M* = 9.550, *SE* = 0.423) showing an overall longer looking time than TD infants (*M* = 8.336, *SE* = 0.394). A significant interaction between condition x group x age group was found. No other interactions were significant, and the random effect was also not significant (*ß* = 1.740, *SE* = 1.102, *z* = 1.578, *p* = 0.114, 95% *CI* 0.503, 6.022). Further inspection of the significant interaction between trial type x group x age group (adjusted for multiple comparisons) revealed that older infants with DS were unable to discriminate the intonation contrast, unlike younger infants with DS and both groups of TD infants. Indeed, only 50% of the older infants with DS showed longer looking times in the switch trials, suggesting that the higher variability in the data of the infants with DS was due to the older age group.

Overall, we found that, like TD infants, infants with DS also exhibit an early ability to discriminate the sentence type intonation contrast in the challenging context of the presence of phonetic variability. However, unlike in TD infants, this early sensitivity to intonation appears to decrease in older infants with DS, as they fail to distinguish between statement and question intonation.

## 5. Discussion

In the current study, we investigated the early perception of intonation in infants with DS to determine whether the development of these prosodic abilities in the first year of life differed between infants with DS and their typically developing peers. Our results demonstrate that infants with DS, like TD infants, also exhibit an early ability to discriminate the intonation contrast between statements and questions in the challenging context of phonetic variability. However, unlike their TD peers, this early sensitivity to intonation appears to decrease in older infants with DS (i.e., in the second half of the first year of life), who fail to discriminate the intonation contrast. This developmental pattern is unexpected in the light of findings on the developmental trajectory of native speech contrasts in typically developing infants, which have shown two main possible paths: an initial sensitivity that is maintained throughout the first year of life (e.g., Polka et al., 2001), or an early lack of/poor sensitivity that is followed by an increase in sensitivity later, in the second half of the first year (e.g., Kuhl et al., 2006). For intonation in particular, typically developing EP-learning infants have been shown to display an initial sensitivity to the statement/question intonation contrast that is maintained (Frota et al., 2014). By contrast, English-learning infants, who are exposed to a language offering a different set of cues to signal the distinction between statements and questions, fail to discriminate the intonation contrast in the first half of the first year of life, but succeed in the second half, thus showing an improvement in their perception abilities (Sundara & Frota, 2025). Thus, the developmental trajectory of EP-learning infants with Down Syndrome is different both from that of typically developing EP-learning infants, who were also perceiving a native contrast, and that of typically developing English-learning infants, who were perceiving non-native, unfamiliar speech stimuli.

From a developmental perspective, it would be expected that infants first recognize sound patterns as different and then learn to match phonetically different patterns into different categories (Liu et al., 2022). The ability to develop sound categories has been argued to support language learning (Werker, 2018). Like TD infants, younger infants with DS demonstrated not only an ability to recognize the intonation contrast, but also an ability to extract and generalize the contrasting intonation patterns across the phonetic variability found in the stimuli. This suggests that, similarly to TD infants, they were able to find what was invariant in speech (for example, the same type of intonation across utterances with different vowels and consonants) and categorize the intonation patterns into two distinct categories (i.e., the ‘statement’ and ‘question’ category). In other words, these results indicate that younger infants with DS might be as able as TD infants to develop prosodic categories.

The finding that infants with DS successfully discriminated utterances that differed only in the intonation cues that distinguish statements and questions during the first half of the first year of life suggests that intonation processing is an initial strength in the very early stages of development in this population. These initial strong discrimination abilities for intonation highlight a crucial developmental window when intonation might be used in early interventions to support language learning. Indeed, infants with DS at these early ages might be able to exploit pitch cues to learn the language, similarly to TD infants. This strongly suggests that prosody, and intonation in particular, which has not been included in early interventions in DS (as shown in recent reviews, Moraleda-Sepúlveda et al., 2022; Seager et al., 2022), should be added as a tool in language and communication interventions from birth.

By contrast, the finding that infants with DS display poor discrimination abilities for intonation after 7 months of age might indicate that older infants with DS are less able to exploit phonetic differences to develop prosodic categories. Consequently, they would be less able to use intonation cues to learn the language (for example, to use pitch cues to facilitate the segmentation of words from continuous speech, or to learn the different sentence types). Indeed, a study on segmentation abilities in atypical development reported that infants with DS from 7 months of age onwards were not able to use intonation as a facilitator for word segmentation, unlike TD infants and infants at-risk for language impairments (Frota et al., 2020). Therefore, for older infants with DS, intonation seems to be a domain of difficulties to be targeted in early intervention, and prosodic/intonation training should be included in language and communication interventions for older infants and toddlers with DS to support their language development.

Why did older infants with DS fail to discriminate the intonation contrast between statements and questions? The present study does not offer an answer to this question. The absence of a looking time difference during the beginning of the habituation phase between older and younger infants with DS, whereas older TD infants show shorter looking times than younger TD infants, seems to suggest that older infants with DS need more attentional resources to process intonation. This might be linked to their decreased sensitivity to the intonation contrast, whereas older TD infants maintain their perception abilities and thus might use less attentional resources with growing age. In typically developing populations, higher attentional demands on language processing have been related with lower language performance (Hula et al., 2007), and toddlers with DS have been shown to exhibit a slow disengagement in attention, which is related to their lower language abilities (D’Souza et al., 2020).

Still, the finding of a different pattern in looking times from older TD infants does not explain why older infants with DS show a diminished ability to discriminate the intonation contrast. The difference in age between the two older groups, with the DS group being older than the TD group, does not seem to provide an explanation either. Although older than the TD group, the older infants with DS showed a similar looking time at the beginning of the habituation phase as the younger infants. Moreover, their older age might even predict a potential improvement in their perception abilities in the test phase, given their longer language experience, instead of a decrease in their perception abilities.

We speculate that the unexpected finding of a decrease in perception abilities for a native intonation contrast in older infants with DS might be related to the perceptual reorganization that characterizes the transition from the first to the second half of the first year of life. This transition has long been described as a key moment for language development due to a perceptual reorganization in the brain that is triggered by sustained language experience (see Werker, 2024, for a recent review). At least part of this perceptual reorganization is related to how a given speech contrast is interpreted. For example, Sato et al. (2010) have shown that, although Japanese-learning infants were able to discriminate a native pitch contrast both at 4 and 10 months of age, their brain responses to the contrast differed: the younger infants seemed to be responding to a phonetic contrast, that is a difference in sound shape only, whereas the older infants were responding to a linguistic contrast, that is they were interpreting the linguistic meaning of the contrast in the language. It might be the case that the older infants with DS display a decreased sensitivity to the intonation contrast because they are struggling to develop distinct prosodic categories and to assign a linguistic interpretation or function to the phonetic distinction. It is important to note that this age period also coincides with observed increases in alterations of the neuronal structure and progressive delays in brain maturation in individuals with DS (Ábrahám et al., 2012; Edgin, 2013; Watson & Meharena, 2023). Future research should try to determine the reasons behind the developmental pattern shown by infants with DS, through experimental paradigms that allow the examination of the characteristics of their brain responses to the intonation contrast (for example, using electroencephalography and/or fNIRS), as well as paradigms that explore whether and when infants are assigning linguistic meaning to the speech contrasts they are exposed to (for example, using versions of the associative learning paradigm, as in Liu and Kager (2018), either implemented with behavioural or brain imaging methods).

A further avenue for future work is the development of age-tailored interventions with a focus on the use of intonation as a tool to support language learning and enhance communication skills in infants with Down Syndrome. In future studies, it will be critical to determine whether age-tailored interventions targeting the sensitive time window for exploring strong pitch discrimination abilities, namely the first 6 months of life, will effectively boost (aspects of) language learning with effects in the second half of the first year, possibly improving discrimination abilities in older infants, as well as having long lasting effects throughout infancy and early childhood.

## 6. Conclusions

This study aimed at examining the early perception of intonation in infants with Down Syndrome, thus contributing to filling the current research gap on early language abilities in individuals with DS. Our findings demonstrated that infants with DS exhibit initial strong discrimination abilities for intonation, similarly to TD infants, and thus might be able to exploit intonation as a facilitator for language learning. However, while TD infants maintain their discrimination abilities during the first year of life, infants with DS show a decreased sensitivity to intonation after 7 months of age, indicating that interventions to support language learning in this population crucially need to take this unexpected developmental path into account. An important limitation of the present study is its inability to explain the reason(s) behind the unexpected decrease in perception abilities for native intonation contrast in older infants. This will be a task for future research, together with additional studies aiming at replicating the current findings and, if possible, including other measures as potential covariates (for example, measures of motor skills and language comprehension and language production measures). Despite these limitations, we highlight the finding that, at the very early stages of development, the perception of intonation is a strength in the language profile of individuals with Down Syndrome, and that language and communication interventions from birth might take advantage of this initial strength.

## Figures and Tables

**Table 1:** Results from the GLLM analysis.

**Fixed Effects**	***F***	***df***	***Denominator df***	***p-Value***
Trial type (same/switch)	30.268	1	38	<0.001
Group (TD/DS)	3.903	1	32	0.06
Age Group (young/older)	0.449	1	30	0.51
Trial type*Group	1.609	1	47	0.21
Trial type*Age Group	2.217	1	46	0.14
Trial type*Group*Age Group	4.268	2	36	0.02
**Analysis of the significant interaction**				
Younger TD infants	*ß* = −3.979, SE = 0.635, *t* = −6.260, *p* < 0.001, *95% CI* −5.242, −2.716			
Older TD infants	*ß* = −2.405, SE = 0.530, *t* = −4.536, *p* < 0.001, *95% CI* −3.455, −1.355			
Younger infants with DS	*ß* = −2.612, SE = 0.953, *t* = −2.742, *p* = 0.008, *95% CI* −4.524, −0.701			
Older infants with DS	*ß* = −1.490, SE = 1.357, *t* = −1.098, *p* = 0.218, *95% CI* −4.261, 1.282			

## Data Availability

Part of the data presented in this study is available in the Supplementary Materials. Further data is available on request from the corresponding author due to privacy or ethical restrictions.

## Supplementary Materials

The following supporting information can be downloaded at https://www.mdpi.com/article/10.3390/ejihpe15100194/s1. Table S1: individual looking time data; Video S1: sample video.
