# Supplementary material for: Early Perception of Intonation in Down Syndrome: Implications for Language Intervention
Source: Eur J Investig Health Psychol Educ. 2025 Sep 26;15(10):194. doi: 10.3390/ejihpe15100194 (PMC12563956; doi:10.3390/ejihpe15100194)
Supplement: Supplementary file 1 [file ejihpe-15-00194-s001.zip › ejihpe-3798387-supplementary-english done.pdf]

Participants	Age Group	Same	Switch
1	Younger	7,320	8,410
2	Younger	2,602	6,036
3	Younger	10,398	10,392
4	Younger	4,750	11,430
5	Younger	3,865	11,927
6	Younger	6,290	7,990
7	Younger	8,956	8,727
8	Younger	12,244	7,161
9	Younger	8,180	13,690
10	Younger	12,450	16,000
11	Younger	5,520	5,360
12	Younger	6,798	10,197
13	Older	5,673	11,685
14	Older	7,962	13,480
15	Older	11,021	10,882
16	Older	9,306	13,577
17	Older	9,250	8,750
18	Older	4,388	15,012
19	Older	10,143	11,340
20	Older	9,657	5,248
21	Older	13,263	10,033
22	Older	8,970	12,209
23	Older	13,781	12,109
